# Use of proton pump inhibitors in scandinavian children and adolescents: An observational study

**DOI:** 10.3389/fped.2023.1052978

**Published:** 2023-02-16

**Authors:** Emilie Raaum Closs, Karl Mårild, Rasmus Gaardskær Nielsen, Ketil Størdal

**Affiliations:** ^1^ Faculty of Medicine, University of Oslo, Oslo, Norway; ^2^ Department of Pediatrics, Sahlgrenska Academy, Gothenburg University, Gothenburg, Sweden; ^3^ Department of Pediatric Gastroenterology, Queen Silvia Children's hospital, Gothenburg, Sweden; ^4^ Hans Christian Andersen Children's Hospital, Odense University Hospital, Odense, Denmark; ^5^ Department of Pediatric Research, University of Oslo, Oslo, Norway; ^6^ Division of Pediatric and Adolescent Medicine, Oslo University Hospital, Oslo, Norway

**Keywords:** gastroesophageal reflux disease, children, PPI, overtreatment, temporal changes, adolescents

## Abstract

**Aims:**

To examine the use of proton pump inhibitors (PPIs) in Scandinavian children with focus on the geographical variation, temporal changes and possible contributing factors to observed changes.

**Methods:**

An observational population-based study of children and adolescents (1-17 years) in Norway, Sweden, and Denmark during the period 2007-2020. Information concerning dispensed PPIs was obtained from the national prescription databases of each country and presented as means per 1,000 children for each country and calendar year in four age categories (1-4, 5-9, 10-13 and 14-17 years).

**Results:**

In 2007, the PPI use in children was similar across Scandinavian countries. An increased PPI use was observed in all countries during the study period, with gradually increasing differences between the countries. In general, Norway showed both the largest total increase and the largest increase in each age category compared to Sweden and Denmark. In 2020 Norwegian children showed, on average, a 59% higher PPI use compared to Swedish children and a more than double the overall dispensation rate than Denmark. In Denmark there was a 19% reduction in dispensed PPIs from 2015 to 2020.

**Conclusion:**

Despite being countries with similar health care systems and without indications of increased incidence of gastroesophageal reflux disease (GERD), we observed considerable geographical variation and temporal changes of PPI use in children. Although this study did not contain data on the indication for PPI use, these large differences across countries and time may indicate a current overtreatment.

## Introduction

Gastroesophageal reflux (GER) is defined as “passage of stomach contents into the esophagus with or without accompanied regurgitation and vomiting”.(1) Gastroesophageal reflux disease (GERD) is first established when the reflux is causing troublesome symptoms or leads to medical complications.(1) GER is physiologic, but the transition to GERD is not clearly defined and depends on clinical judgement. The prevalence of GERD in childhood varies between 2%–8%, depending on its definition, study design and age group (2, 3).

Proton pump inhibitors (PPIs) are considered first-line medical treatment for GERD (4). PPIs are a class of medications that selectively inhibit the gastric proton pump in the parietal cell, leading to reduced acid secretion and thereby increasing the pH (5). Prior to the emergence of PPIs, H_2_-receptor antagonists (H_2_As) were the main medical treatment for GERD. These drugs lower gastric acidity by competitively inhibiting histamine from binding to H_2_-receptors on parietal cells (5). Although rapid onset, they are considered overall less effective than PPIs (6, 7).

Over the last decades, there has been a development in both the diagnostic approach to and the use of PPIs to control symptoms of GERD due to several factors discussed later. Among these, changes of the 2009 to 2018 edition of the European and North American Societies of Gastroenterology, Hepatology and Nutrition (NASPGHAN/ESPGHAN) guidelines with a tendency to a more liberal use of time limited PPI trials as a diagnostic tool for children above 1 year, are likely of major importance (8, 9). While generally considered a safe drug, there are increasing concerns that PPI use, in particular long-term treatment, may increase the risk of several adverse effects including changes in the gut microbiome, infections and fractures (10–12).

The aim of this study is to examine the use of PPIs in children and adolescents (aged 1-17 years) in the Scandinavian countries during the period 2007-2020 with focus on the geographical variation and temporal changes in PPI use.

## Methods

This is an observational study covering the PPIs dispensed in the Scandinavian countries Norway, Sweden, and Denmark, during the period 2007-2020 and ages 1-17 years. The Anatomical Therapeutic Chemical code (ATC) for PPIs is ATCA02BC and ATCA02BA for H_2_As. These drugs are available for these age groups by prescription only, and therefore registered in the nation-wide prescription registries. The use of PPIs in infants (<1 year) is in general not recommended by the international guidelines (4, 8), and not approved for use before 1 year in the Scandinavian countries and European Union (13–16). For those reasons, infant PPI use was not examined in this study, but dealt with in another separate study (17).

### Prescription data

A formal approval was granted from the national prescription databases of Norway (18), Sweden (19), and Denmark (20) to gain access to relevant aggregated data concerning the use of PPIs among children and adolescents. All registers contain information concerning all prescription drugs sold in each specific country. Information about H_2_A- use was collected based on publicly available data obtained from the same sources for Norway and Sweden. Corresponding material was not available for Denmark. Although not fully equivalent concerning age groups (0-19 years for H_2_A), these data were considered useful for comparing temporal trends. In addition, population data was obtained from Statistics Norway (21) because this information was not included in the original data set.

### Analysis

All data sets were based on dispensed prescriptions at pharmacies and were calculated as the number of prescriptions per 1,000 children for each country and calendar year. The data for each country concerning PPIs, were then divided into four age categories (1-4, 5-9, 10-13 and 14-17 years) and presented as prescriptions per 1,000 children per year. The use of H_2_As per 1,000 children per year were also obtained at the start and end of observation (2007 and 2020) and compared with the use of PPIs.

### Ethics

Because this study was based on aggregated, anonymous data that could not be linked to any individual, no ethical approval or informed consent was needed (22).

## Results

In 2007 (first year of data capture), the use of PPIs was similar in the three countries at around 10-11 dispenses per 1,000 children per year. The use of PPIs increased in all three countries during the period 2007-2020 (Table 1), but there were major differences in the time trend across countries.

**Table 1:** Population and dispensed proton pump inhibitors and H_2_-receptor antagonists for the period 2007-2020 by age and country.

	Sweden	Denmark	Norway
**POPULATION 2007**			
1 to 4 years	411 927	260 006	228 710
5 to 9 years	472 130	333 694	289 014
10 to 13 years	430 457	282 442	241 112
14 to 17 years	511 256	272 691	241 534
**Dispensed PPIs in total**	19 317	11 780	10 471
Dispenses/1000/year PPI	10.6	10.3	10.5
**POPULATION 2020**			
1 to 4 years	483 911	247 813	225 138
5 to 9 years	622 220	301 910	297 612
10 to 13 years	496 150	270 755	242 205
14 to 17 years	468 190	270 857	228 710
**Dispensed PPIs in total**	44 189	17 545	33 736
Dispenses/1000/year/PPI	21.3	16.1	34.0
Dispensed H_2_As* **in total**	2803	Na**	2355
Dispenses/1000/year H_2_A*	1.2	Na**	2.0

*2019 was used for H_2_Ás due to the marked decrease after Ranitidine was withdrawn from the market in 2019/2020. The material also concerned ages 0-19.

**Na = Not available.

**Figure 1:**
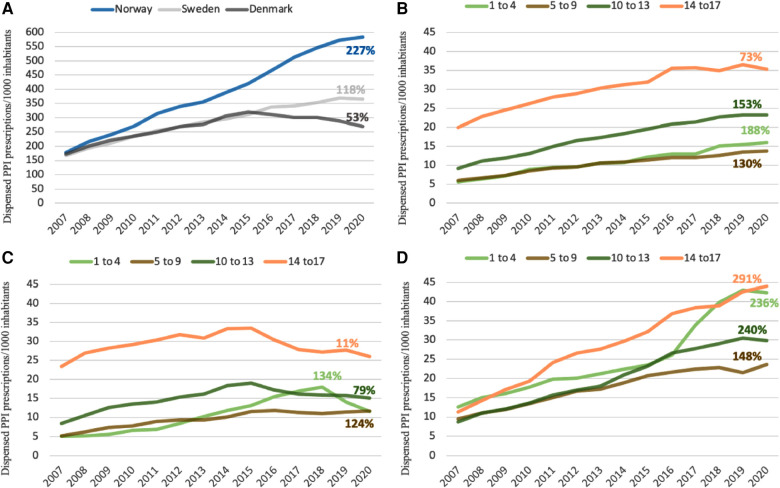
(**A–D**): temporal changes of PPIs by age and country. Percentages showing the increase in dispensations of PPIs for each age group and each country from 2007 to 2020. A = All countries, B = Sweden, C = Denmark, D = Norway.

During the study period, Norway had the largest total increase compared to Sweden and Denmark, with dispenses increasing from 10.5 to 34.0/1000/year. In Sweden childhood PPI use increased steadily from 10.6 to 21.3 per 1,000 children/year and in Denmark from 10.3 to 16.1 per 1,000 children/year. In 2020, Norway had a 59% higher overall dispensation rate than Sweden and a more than double the overall dispensation rate than Denmark. As shown in Figures 1A–C, Denmark was the only country where we noted a decrease in childhood PPI use in later years. The use increased until 2015, but from 2015 to 2020, the overall dispensation rate decreased by 19%. In the same period, the total number of dispensations of PPIs continued to increase by 39% in Norway and 18% in Sweden.

For comparison, from 2007 to 2020, the use of H_2_A decreased from 2.9 to 1.2 dispensations per 1,000 children/year in Sweden and was largely unchanged in Norway (1.8 and 2.0 dispensations per 1,000 children/year, respectively) (Table 1).

Between 2007 and 2015, we noted an even increase in PPI use across all age groups in all three countries (Figures 1B–D). However, since 2015, we noted a considerable decrease in PPI use in Danish adolescents (14-17 years of age) and, in contrast, a marked increased PPI use in Norwegian children aged 1-4 years reaching the same rate as Norwegian children aged 14-17 years (2020: 42 and 44 per 1,000 children, respectively). In Denmark and Sweden, the number of dispensations was highest in the oldest age group during the whole study period.

## Discussion

The main findings in this study were a marked increase in the number of dispensations of PPIs in all the Scandinavian countries from 2007 until 2020. However, while childhood PPI use showed a steady increase in Norway and Sweden during this time period, there was a noticeable reduction in Denmark from 2015 until 2020. The most considerable increase was found in Norway.

The causes behind an increase in the number of dispensations of a specific group of pharmaceutical agents such as PPIs is likely multifactorial rather than caused by one single factor. There are no data indicating a rise in GERD in Scandinavian children over the past two decades that could explain our findings. Also, there are to our knowledge no reasons to believe there is a true difference in the prevalence of childhood GERD across Scandinavian countries. Access to the relevant medication and formulations suitable for children is likely of major importance. Since 2014, Norwegian children may be prescribed subsidized PPIs by any physician (rather than by pediatricians only) and without any required specific diagnostic procedures (23). PPIs as a granulate formulation was licensed in 2008, These changes in access might partly explain the particular increased PPI dispensations in the age group of 1-4 years after 2015.

A concomitant reduction in the number of dispensations of H_2_As was observed in Sweden but not in Norway. The reduction in dispensed H2A was too limited to explain the marked increase in PPI dispenses.

There is increasing evidence of potential adverse effects with particularly long-term PPI use, illustrating the importance of weighing potential benefit against harms when prescribing PPIs to children. Children exposed to PPIs have been reported to have changes in the microbiome and increased risk of gastrointestinal infections including Cl. Difficile (12). Furthermore, an increased risk of allergic diseases and obesity after exposures the first two years of life are of concern (24). Reduced absorption of minerals may also impact on bone health by similar mechanisms as in adults (11). Furthermore, it has been reported that use of PPIs over several weeks increase the risk of rebound hyperacidity. This is thought to be due to a reflective increase in gastrin secretion secondary to the PPI induced hypoacidity (25, 26). Rebound hyperacidity may cause dyspeptic symptoms itself, which may lead to the reinstitution of medication and thereby a vicious cycle that prevents stopping PPI treatment. In addition, hypergastrinemia might be associated with an increased risk of developing gastric neuroendocrine tumors (NET) (27). The clinical relevance of these tumors in young people and the strength of the associations are however controversial.

To the best of our knowledge, our study is the first to examine the use of PPIs for the purpose of comparison across children of several countries over time. A major strength in our study is therefore the opportunity to evaluate similarities, differences, and trends between countries. In Table 2 we summarize studies of PPI use in single countries. These studies indicate increasing use of PPIs over time.

**Table 2:** Studies concerning the trends in the use of PPIs in children and adolescents.

Reference	Study design	Population	Study period	Main findings
S P Nelson, the United States, 2009 (28)	Cohort study	0–18 years	1999–2005	Incidence of diagnosed GERD increasing from 3.4%–12.3% during the study-period. In addition, proportion of PPI-initiated patients nearly doubled, from 31.2%–62.6% of all diagnosed.
Ruigómez, the United Kingdom, 2011 (29)	Retrospective cohort study	1–17 years	2000–2005	Of 1,700 patients with GERD, initially 49.2% were prescribed antacids. Similar proportions achieved H2A (23.3%) and PPI (22.9%), but 24.7% of those given H2A, switched to PPIs. The use of PPIs increased with age and during study period.
De Bruyne, Belgium, 2014 (30)		0–16 years	1997–2009	The monthly volume of all reimbursed anti-reflux medicines increased seven-fold during the study period. Most extensive increase in the prescription of PPIs
Quitadamo, Italy, 2014 (31)	Prospective study	100 randomized Italian pediatricians	2012–2013	Only 2% showed adherence to guidelines. 57% prescribed PPIs to children <8-12 without further examination. Overall rate of pediatricians overprescribing was estimated to 79%
Aznar-Lou, Denmark, 2019 (32)	Register-based nationwide study	0–17 years	2000–2015	Total annual use of PPIs increased eight times during the study period, while prevalent users increased from 0.1-3.1/1,000 and new users increased from 1.2-8.0/1000
Abrahami, the United Kingdom, 2020 (33)	Population-based cross-sectional study	Nationwide, both pediatric and adult population	1990-2018	PPI prevalence increased from 0.2%–14.2% of the population in total during the study period. Separate data for children were not available
Arnoux, France, 2022 (34)	Single-center, observational, retrospective study	0–18 years	2019	11% of the hospitalized children during the 6-month study period were given PPIs in different hospital departments. Only 34.5% were according to applicable guidelines.
Yang, France, 2022 (35)	A Time-Series Analysis	Age subgroups, < 2 years, 2–11 years, and 12-17 years	2009–2019	Mean PPI prescription rate of 52.5 per 1,000 inhabitants per year. Prescription rate increased 41% in the overall pediatric population during the study period (+110% in infants). Significant decrease in adolescents only after the release of international guidelines.

Another strength is that the data sets are large and comprehensive based on nation-wide population-based registers avoiding any selection bias. Available data from three countries with similar populations and public health care, made it possible to study unexplained variation in prescription practice. The study period 2007-2020 is interesting in itself due to the extensive research concerning the use and possible side effects of PPIs among children and adolescents. In the same period, NASPGHAN/ESPGHAN published the first guidelines for the handling of GERD in children and adolescent in 2009, which were revised 2018 (8, 9). Our study also has limitations.

We lacked data on defined daily doses of PPI. To obtain such information, individual data are required after legal approvals, and would be strengthened by linkage to patient registers for diagnoses and procedure codes. The material does not contain information concerning patient´s adherence to the pharmacological treatment dispensed. As a result, our data gives information concerning the number of dispenses, but not whether the patients take their medicines or not. Although adherence to prescriptions might have changed some during the study period, and maybe across borders, this is not likely to be a main cause of any changes observed in the number of dispenses of PPI. Another limitation to our study is that we are not able to describe whether the size of each prescription has changed, which might have an impact on how we would interpret our results. In addition, the data sets do not distinguish between different PPIs approved for children and adolescents or give any option to distinguish on potential sex differences.

It would also add important information to examine to what extent PPIs are prescribed by non-specialists compared to pediatricians. Without supplementary investigations that are available only in specialized care, it may be difficult to differentiate between GER and GERD. This differentiation may be even more challenging if the examining doctor meets children with suggestive symptoms quite infrequently. Reflux is physiologic in infants and occurs frequently also in older children and adolescents (2). Without any pathognomonic symptoms or gold standard diagnostic tool, it is challenging to strictly differentiate normal physiology from disease. Lack of precise diagnostic criteria is likely to open for large variation in clinical practice and may lead to overdiagnosis and overtreatment. In our study covering Scandinavian countries with similar health care systems, the marked differences in childhood PPIs use over time and geography noted in this study, is likely to be a sign of overtreatment (i.e., unsubstantiated use).

PPIs are also use for other conditions than suspected GERD. Functional dyspepsia is frequent in older children and adolescents. Based upon clinical examination alone, differentiating GERD from functional dyspepsia may be difficult. As a definitive diagnosis may require invasive procedures as gastroscopy, esophageal biopsies and 24 h pH metry with or without impedance, the application of a “treat” instead of “test” strategy may partly explain increasing PPI use in older children. Information regarding diagnostic procedures was not available from our datasets, and beyond the scope of this study.

In typical cases with symptoms like regurgitation, heartburn and pain in the chest or upper abdomen, a preliminary diagnosis is based on history and clinical examination alone. Despite this, neither the 2009 nor the 2018 editions of the NASPGHAN/ESPGHAN guidelines recommend long-term use of PPIs without further examination. Nevertheless, there are some differences important to be aware of. The 2009 guidelines recommended a “time-limited” trial of maximum 4 weeks for older children and adolescents, without any specific age limit downwards. The 2018 guidelines recommend a 4–8-week trial for children and adolescents with typical symptoms, but makes a clearer recommendation against empiric use in infants (8, 9). The 2018 recommendation of a prolonged (from 4 to 4-8 weeks) use of PPIs is parallel to our findings of marked increase in the number of dispensations in the youngest age group over the last years of study.

It is important to acknowledge that untreated GERD will affect children and adolescents in negative ways, both short-term and long-term. Frequent regurgitation, and accompanying symptoms like abdominal pain, heartburn, halitosis, and persistent coughing may reduce the quality of life for those affected. If the reflux is severe and left untreated for a long time, it might result in esophagitis and secondary strictures. A dreaded complication of chronic (>5 years) or frequent (>1 weekly) GERD is the development of Barret´s esophagus, a risk factor for esophageal cancer in late adulthood (36). The balance in clinical practice will always be to avoid missing the diagnosis of GERD and at the same time to avoid unnecessary long term use of PPIs in children without a defined diagnosis.

## Conclusion

In Scandinavian children aged 1-17 years PPI use has increased considerably from 2007 to 2020. We observed marked geographical variation and temporal changes of PPI use in children in three neighboring countries with similar health care system and without indications of differences in incidence of gastroesophageal reflux disease (GERD). Although factors such as more use of time-limited PPI trials as a diagnostic tool for children may contribute, the marked differences across countries and time may indicate a current overtreatment.

## Data Availability

The data analyzed in this study is subject to the following licenses/restrictions: The datasets were provided by the national prescription databases and are available from the data owners. Requests to access these datasets should be directed to; https://www.reseptregisteret.no, https://www.socialstyrelsen.se/en/statistics-and-data/registers/national-prescribed-drug-register/, https//sundhedsdatastyrelsen.dk/da/registre-og-services/om-de-nationale-sundhedsregistre/sygedomme-laegemidler-og-behandlinger/Laegemiddelstatistikregisteret.
